# Therapeutic Management of Septic Venous Thrombosis: A Narrative Review

**DOI:** 10.3390/idr18020031

**Published:** 2026-04-03

**Authors:** Anabel Franco-Moreno, Ana Bustamante-Fermosel, Juan Torres-Macho, Belén Comeche-Fernández

**Affiliations:** 1 Venous Thromboembolism Unit, Hospital Universitario Infanta Leonor, 28031 Madrid, Spain; 2 Department of Internal Medicine, Hospital Universitario Infanta Leonor, 28031 Madrid, Spainbelen.comeche@salud.madrid.org (B.C.-F.); 3 Faculty of Medicine, Universidad Complutense de Madrid, 28040 Madrid, Spain; 4 Infectious Disease Unit, Hospital Universitario Infanta Leonor, 28031 Madrid, Spain

**Keywords:** venous thrombosis, sepsis, antimicrobial treatment, anticoagulant therapy, infectious complications, recanalization

## Abstract

Background/Objectives: Septic venous thrombosis is an uncommon complication but clinically significant due to its high morbidity and mortality and the complexity of therapeutic decision-making. The lack of standardized guidelines and the scarcity of high-quality studies complicate clinical management, as most available evidence derives from highly heterogeneous case series and retrospective studies. In this context, a comprehensive overview is essential to guide real-world practice. Methods: This manuscritp provides an in-depth review of the treatment of septic venous thrombosis at its most frequent sites, including the portal vein and its branches, the pelvic veins, catheter-associated events, the internal jugular vein, and dural venous sinus thrombosis. Results: Across all scenarios, early initiation of appropriate antibiotic therapy is the cornerstone of treatment and must be tailored to the suspected source of infection and the patient’s clinical course. In parallel, although the role of anticoagulation remains debated, several observational studies suggest potential benefits in terms of recanalization and complication prevention, particularly in selected patients. Conclusions: However, the decision to anticoagulate should be carefully individualized within a multidisciplinary framework. Despite the recent progress, many clinical uncertainties remain. Therefore, well-designed clinical trials are needed to define optimal therapeutic strategies for this condition.

## 1. Introduction

**Figure 1:**
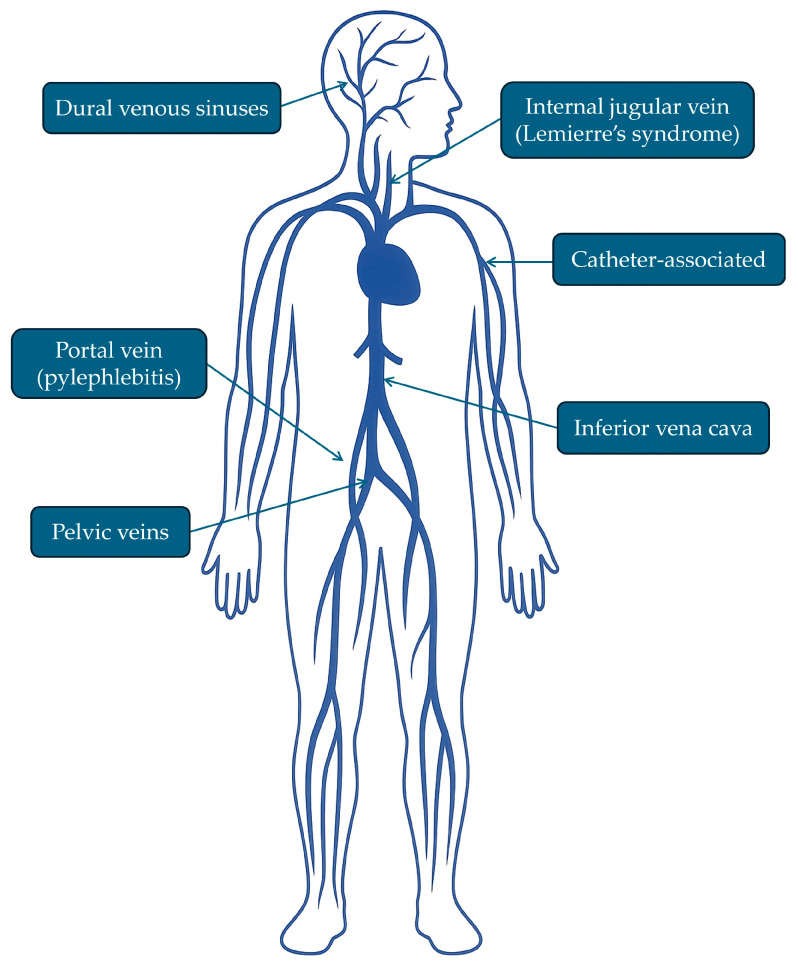
Most frequent locations of septic venous thrombosis.

Septic venous thrombosis (SVT) is defined as thrombus formation in the setting of an infectious process—most commonly bacterial or fungal—with or without bacteremia and may involve the vascular wall as well as adjacent perivascular tissues. The most frequent locations include the portal vein and its branches, catheter-associated thrombosis, pelvic veins, dural venous sinuses, and the internal jugular vein (Figure 1). Although SVT is uncommon, it is associated with substantial morbidity and mortality, particularly among immunosuppressed patients, those with severe sepsis, or those with delayed diagnosis, with mortality rates exceeding 10% in some series [1,2,3].

Diagnosis is challenging because the clinical presentation is often nonspecific, requiring a high index of suspicion and confirmation with imaging studies. This challenge is compounded by the lack of standardized guidelines and limited evidence regarding optimal antibiotic duration, the role of anticoagulation, and the most effective combination of therapeutic strategies.

Although inflammatory markers such as C-reactive protein, procalcitonin, and leukocyte count are commonly used to assess severity in sepsis, their specific role in predicting outcomes in septic venous thrombosis remains unclear. Available data are extrapolated from general sepsis populations and do not allow site-specific conclusions.

This review addresses pathophysiologic mechanisms, microbiology, clinical manifestations, diagnostic approaches, and therapeutic options for SVT, with particular emphasis on antimicrobial therapy and anticoagulation. The aim is to provide an updated, comprehensive perspective to support clinical decision-making and promote standardized management of this condition.

## 2. Methods

This manuscript is a narrative review that provides a comprehensive, clinically oriented overview of the therapeutic management of septic venous thrombosis across different anatomical locations. A literature search was conducted in the following electronic databases: PubMed/MEDLINE, Embase, and Scopus. In addition, reference lists of relevant articles were manually screened to identify further pertinent publications. The search primarily focused on studies published between January 2000 and December 2025, with particular emphasis on the most recent literature. Older references were included selectively when considered seminal or essential for understanding the pathophysiology or historical evolution of septic venous thrombosis management. Search strategies combined Medical Subject Headings (MeSH) terms and free-text keywords related to septic thrombosis and its management. The main terms included “septic venous thrombosis”, “septic thrombophlebitis”, “pylephlebitis”, “Lemierre syndrome”, “cavernous sinus thrombosis”, “catheter-related septic thrombosis”, “antimicrobial therapy”, “antibiotic treatment”, “anticoagulation”, “heparin”, and “direct oral anticoagulants”. These terms were adapted as appropriate for each database.

Articles were selected based on clinical relevance, quality of evidence, and applicability to real-world practice. Priority was given to clinical guidelines, observational studies, and extensive case series addressing therapeutic strategies, antimicrobial regimens, and the role of anticoagulation in septic venous thrombosis. Case reports were included when they provided valuable information on rare locations, emerging therapies, or uncommon clinical scenarios. No formal exclusion criteria were applied, consistent with the narrative nature of the review. Although no formal risk-of-bias assessment tool was applied, studies were critically appraised based on methodological quality, consistency of findings, and clinical relevance.

A total of 1766 records were initially identified. After removing 898 duplicates, three reviewers (A.F.M., A.B.F., and B.C.F.) independently screened the titles and abstracts of all identified records to assess their eligibility based on relevance and clinical applicability. The full texts of potentially relevant articles were retrieved and evaluated in detail. Reviewer disagreements during the selection process were resolved through discussion and consensus; if consensus could not be reached, a fourth reviewer (J.T.M.) was consulted to make the final decision. To ensure consistency and reproducibility in the selection process, all reviewers used a standardized screening form. Following screening and eligibility assessment, 60 studies were ultimately included. No language restrictions were applied.

## 3. Coagulation Abnormalities in Sepsis

Sepsis results from a dysregulated host response to infection. In most individuals, the inflammatory response is adaptive and contributes to infection control; however, in sepsis, this response becomes imbalanced, with increased proinflammatory activity and reduced anti-inflammatory counterregulation. Among other consequences, this triggers a procoagulant and antifibrinolytic state. In sepsis, multiple cell lineages—including monocytes, macrophages, and neutrophils—release cytokines such as tumor necrosis factor alpha (TNF-α), interleukin-1 (IL-1), and interleukin-6 (IL-6) [4]. These mediators damage the endothelium, which expresses tissue factor (TF), thereby activating the extrinsic coagulation pathway, leading to thrombin generation and subsequent fibrin deposition in the microcirculation [4]. This effect is amplified by fibrinolysis inhibition mediated by the massive release of plasminogen activator inhibitor-1 (PAI-1), along with platelet activation [5]. Simultaneously, sepsis impairs endogenous anticoagulant mechanisms, including antithrombin, activated protein C, and the tissue factor pathway [6]. The combination of coagulation activation and suppression of anticoagulant mechanisms predisposes to severe complications, such as disseminated intravascular coagulation (DIC), characterized by clot formation in small- and medium-sized vessels and the potential to cause multiorgan damage [7].

Importantly, antithrombin levels are associated with outcome in sepsis, but their predictive value is limited and context-dependent. Lower antithrombin activity is consistently observed in non-survivors compared to survivors in both adult and pediatric sepsis cohorts, and lower levels are associated with an increased risk of organ dysfunction and mortality, particularly in sepsis-associated DIC [8,9,10]. However, antithrombin activity alone has only limited discriminative power for mortality prediction and is inferior to established clinical scoring systems such as SOFA or APACHE III [8].

In clinical practice, D-dimer, platelet count, prothrombin time (PT), and viscoelastic hemostatic assays (VHAs) are the most useful laboratory analyses for identifying patients with sepsis at increased risk of developing venous thrombosis. Elevated D-dimer reflects increased fibrin formation and breakdown and is associated with hypercoagulability and the risk of thrombosis in sepsis [11,12,13]. Thrombocytopenia and prolonged PT are markers of sepsis-induced coagulopathy and correlate with disease severity and risk of thrombotic complications [14]. VHAs, such as thromboelastography and rotational thromboelastometry, provide real-time assessment of clot formation, strength, and lysis and can detect hypercoagulable states and impaired fibrinolysis, both of which are linked to an increased risk of venous thrombosis in septic patients [15,16]. Early stages of sepsis-associated coagulopathy often show hypercoagulability on VHAs, preceding overt DIC and thrombotic events [7,8]. Emerging biomarkers, including endothelial-derived microparticles, have shown promise in predicting microvascular thrombosis and DIC but are not yet widely available for routine clinical use [17]. Combined assessment of coagulation and inflammatory markers (e.g., D-dimer and PT) improves risk stratification for progression to septic shock and thrombotic complications.

Some microorganisms or their toxins exert a direct prothrombotic effect. Lipopolysaccharide (LPS) from Gram-negative bacteria can induce the contact pathway by activating factors such as factor XII, factor XI, and prekallikrein, accelerating thrombin formation, as shown in experimental models of *Escherichia coli* sepsis [18]. In addition, LPS stimulates TF expression in monocytes and endothelial cells, thereby promoting the extrinsic coagulation pathway [19]. *Staphylococcus aureus* secretes proteins such as coagulase (Coa) and von Willebrand factor-binding protein (vWbp), which can activate prothrombin and generate thrombin, thereby facilitating fibrin formation [20]. *Fusobacterium* spp., particularly *Fusobacterium necrophorum*, produce endotoxins and hemagglutinins that promote platelet aggregation and endothelial injury, favoring TF expression and thrombosis [21]. *Bacteroides fragilis* possesses polysaccharide capsules and lipooligosaccharides that induce platelet activation and TF expression and have been linked to intra-abdominal septic thrombosis [22]. Finally, porins from *Salmonella typhimurium* directly promote coagulation by accelerating thrombin activity, an effect observed experimentally in vitro [23].

A recent study provides a comprehensive analysis of the evolution and limitations of clinical trials evaluating anticoagulation in sepsis. Early studies investigating anticoagulant strategies initially suggested survival benefits. However, these findings were not confirmed in subsequent trials [24].

## 4. Sepsis and Venous Thrombosis

Sepsis increases the risk of venous thrombotic events [25]. This phenomenon has gained particular relevance in recent years, in part due to SARS-CoV-2 infection. Since the start of the COVID-19 pandemic, multiple studies have documented a predisposition to venous thrombotic complications [26,27]. Although the mechanisms linking sepsis to venous thrombosis are not as well defined as those in arterial thrombosis, sepsis-related hypercoagulability, together with immobilization, dehydration, hypoxia, and the presence of venous catheters, contributes to this risk [28,29]. Consistent with this observation, the Surviving Sepsis Campaign (SSC) recommends prophylaxis with low-molecular-weight heparin (LMWH) to prevent venous thromboembolism in patients with sepsis, unless contraindicated [30].

## 5. Septic Venous Thrombosis at Specific Sites

### 5.1. Portal Vein and Its Branches

Pylephlebitis, defined as septic thrombosis of the portal venous system, is a severe complication of intra-abdominal infections. Historically, it was associated with appendicitis and intra-abdominal abscesses; however, diverticulitis and biliary infections—particularly cholangitis—are now the most frequent causes [2]. Reliable population-level incidence estimates are lacking, although a recent systematic review estimated an incidence of 0.3–2.7 cases per 100,000 person-years [2]. Diagnosis requires radiologic confirmation of portal vein thrombosis. Ultrasound, particularly with Doppler, is an accessible and valuable tool for initial detection. In contrast, computed tomography (CT) and magnetic resonance imaging (MRI) provide more detailed characterization and are more sensitive and specific [2,31]. Despite diagnostic and therapeutic advances, mortality associated with pylephlebitis remains substantial, at around 10–15% in recent series [2]. The most commonly implicated microorganisms are *Bacteroides* spp., *Escherichia coli*, and *Streptococcus* spp. [1,32]. Approximately 30% of cases are polymicrobial. Notably, *Bacteroides* spp. have been associated with a higher thrombogenic potential [1,32].

No clinical trials have defined the optimal antibiotic regimen or duration for pylephlebitis. Given the initial severity, intravenous treatment is recommended until clinical stability is achieved, after which an oral step-down therapy may be considered. The regimen should be tailored to the underlying source. Suggested options include a third-generation cephalosporin or a fluoroquinolone combined with metronidazole, or monotherapy with beta-lactam/beta-lactamase inhibitor combinations or carbapenems, depending on the individual risk for multidrug-resistant organisms [2]. Duration is also not clearly established; in patients with favorable clinical evolution, therapy is commonly maintained for 4 to 6 weeks, using a fluoroquinolone plus metronidazole during the oral phase [2,32].

There are no randomized controlled trials definitively supporting anticoagulation in pylephlebitis, and most evidence comes from small, low-quality observational studies. The main goal of anticoagulation is to prevent thrombus progression and promote resolution. Although routine anticoagulation is not universally recommended, it may be considered in selected patients. Clinical scenarios that may justify anticoagulation include thrombus progression on serial imaging, persistent fever or bacteremia despite appropriate antibiotics, thrombotic extension beyond the portal vein—especially into the mesenteric vein—and the presence of an underlying hypercoagulable state [2]. Anticoagulation in pylephlebitis has been associated with higher portal vein recanalization rates and a lower incidence of complications related to chronic portal hypertension. Evidence is limited to retrospective case series. A retrospective study including 67 pylephlebitis cases over 19 years reported a significantly higher portal recanalization rate in anticoagulated versus non-anticoagulated patients (58% vs. 21%, respectively; *p* < 0.05) [31]. Chronic symptoms attributable to portal hypertension were also substantially lower in the treated group (11% vs. 47%; *p* < 0.05). Similar findings were reported by Choudhry et al. [33]. Another study analyzing 100 cases of pylephlebitis in non-cirrhotic patients found higher complete recanalization rates among anticoagulated patients and lower overall mortality (6% vs. 22%) [1]. In this setting, direct oral anticoagulants (DOACs) may represent an option. Likewise, Baril et al. reported that among 32 pylephlebitis cases not receiving anticoagulation, five died, compared with none among 12 anticoagulated patients [34].

Clear recommendations regarding the anticoagulation strategy are lacking. In practice, therapy is often initiated with LMWH and then transitioned to an oral anticoagulant. Although not derived from septic portal thrombosis, two clinical trials have evaluated rivaroxaban in non-cirrhotic portal vein thrombosis. Plessier et al. demonstrated a significant reduction in recurrence or progression without an increase in major bleeding [35]. Concordant results were reported by Ageno et al., with favorable recanalization rates and a safety profile comparable to conventional anticoagulation [36]. Although favorable outcomes with apixaban have been reported, experience remains limited [37].

The optimal duration of anticoagulation is not established and should be individualized based on clinical and radiologic evolution. In general, 3 months is commonly recommended [38]; however, portal vein recanalization may occur within 2 to 4 weeks, suggesting that shorter courses may be feasible in selected cases. Additional procedures such as thrombolysis and percutaneous techniques—including catheter aspiration of thrombus or pus and intraportal antibiotic infusion—have been described in isolated cases [39].

### 5.2. Pelvic Veins

Septic pelvic thrombophlebitis typically occurs postpartum and is frequently associated with endometritis. However, it may also be observed in pelvic inflammatory disease, gynecologic surgery, estrogen use, or patients with cancer [40]. Two primary forms are described: ovarian vein thrombosis (the more common form) and deep pelvic venous thrombosis. The condition should be suspected in women who develop persistent fever and abdominal pain during the first postpartum week. Diagnosis is established by imaging; contrast-enhanced CT in the venous phase or MRI with gadolinium-enhanced venography are considered the modalities of choice [40].

The main microorganisms implicated in septic pelvic thrombophlebitis include *Streptococcus* spp., enteric Gram-negative bacteria, and anaerobes, although cultures are negative in most cases [41]. Therefore, empiric regimens should cover these pathogens. Historically, regimens used for endometrial infections, such as gentamicin plus clindamycin, have been employed [40,42,43]. However, it is also reasonable to adopt intra-abdominal infection approaches, either monotherapy with beta-lactams or combinations of cephalosporins or fluoroquinolones with an anaerobe-active agent [41]. In this context, clindamycin is considered safer than metronidazole during breastfeeding. In patients with sepsis or hemodynamic instability, toxin-mediated disease should be considered; thus, in addition to a beta-lactam or fluoroquinolone, linezolid may be included, with clindamycin as a valid alternative. Regarding duration, most reports recommend continuing antibiotics for 48–72 h after fever resolution [40,42,43,44].

The role of anticoagulation in septic pelvic thrombophlebitis remains debated. When combined with antibiotics, it may limit thrombus progression into the inferior vena cava and reduce the risk of embolization. Available evidence primarily comes from observational studies and case series. In the most extensively published series (46 patients treated with heparin), 91% achieved clinical resolution within the first week, with no documented recurrences or hemorrhagic complications [45]. Reported regimens include unfractionated heparin (UFH) and LMWH, with transition to oral anticoagulants in some cases. Optimal duration is not defined and is typically individualized. For thrombosis limited to pelvic venous branches, a minimum of 2 weeks is often recommended. In contrast, for ovarian vein involvement, iliac vein or caval extension, or in the presence of septic emboli, anticoagulation is usually extended to at least 6 weeks [40]. Most of the available data are derived from observational studies.

### 5.3. Catheter-Associated Venous Thrombosis

Catheter-associated septic venous thrombosis, or suppurative thrombophlebitis, is defined as the coexistence of device-related thrombosis and bacteremia (therefore, this discussion does not address catheter-related infection itself). It is a serious complication of intravascular device infections. Management typically combines early catheter removal, antibiotics, and, in most cases, anticoagulation.

The most prevalent microbiologic etiologies include *Staphylococcus aureus* and coagulase-negative staphylococci (CoNS), *Streptococcus* spp., enteric Gram-negative bacteria, *Pseudomonas aeruginosa*, and *Candida* spp. [46,47]. Given the risk of hematogenous metastatic infection, particularly if the catheter is not removed, prompt empiric antibiotic therapy covering these etiologies is recommended, with consideration of the possibility of multidrug-resistant organisms [47,48,49]. For Gram-positive coverage, vancomycin (teicoplanin is not recommended due to reported CoNS resistance) or daptomycin is advised until microbiologic results are available [47]. When these agents are unavailable, linezolid may be considered, although it is not recommended in severely ill patients. In the presence of risk factors for Gram-negative bacilli (unstable patients, femoral catheter placement, immunocompromised status, Gram-negative colonization, or prolonged ICU stay), coverage against these pathogens should be added, including consideration of multidrug resistance based on local epidemiology [47,48,49]. Options include piperacillin–tazobactam, carbapenems, fourth-generation cephalosporins, fluoroquinolones, or aminoglycosides. If *Candida* infection is suspected (e.g., patients receiving parenteral nutrition, oncology patients, prolonged antibiotic exposure, femoral catheter placement, or *Candida* colonization), empiric antifungal therapy is recommended.

Regarding duration, Infectious Diseases Society of America (IDSA) guidelines previously recommended 3 to 6 weeks of antibiotics; however, more recent studies suggest that courses shorter than 21 days are safe [47,50].

An additional scenario is thrombophlebitis associated with injection drug use. In these patients, beyond the considerations above, given the risk of premature hospital discharge that may prevent completion of an optimal antibiotic course, empiric use of long-acting antibiotics such as the lipoglycopeptides dalbavancin or oritavancin may be considered [51].

A controversial aspect is the optimal timing of catheter removal. Early removal is generally considered safe. However, some authors have proposed delaying removal in the presence of large thrombi adherent to the catheter tip by initiating anticoagulation first, although this approach lacks robust evidence [48].

The role of anticoagulation in catheter-associated septic venous thrombosis remains ill-defined, with heterogeneous evidence mainly derived from observational studies. A small prospective cohort study found no differences in relapse or mortality rates at 12 weeks between patients who did and did not receive anticoagulation [52]. In contrast, a retrospective US study found a lack of anticoagulation to be an independent predictor of poor outcomes [46]. IDSA guidance acknowledges that the role of anticoagulation in this setting is uncertain and highlights the need for clinical trials to define any potential benefit [53] more precisely.

### 5.4. Internal Jugular Vein

Septic thrombophlebitis of the internal jugular vein, known as Lemierre syndrome, is characterized by venous wall inflammation, formation of an infected thrombus, involvement of adjacent soft tissues, bacteremia, and, in some cases, septic emboli [54]. It usually follows an oropharyngeal infection but has also been described in association with odontogenic infections, mastoiditis, otitis media, sinusitis, or parotitis [54]. Etiologic agents typically correspond to the normal oropharyngeal flora, with *Fusobacterium necrophorum* being the most frequently implicated anaerobe. However, other *Fusobacterium* spp., enteric Gram-negative bacteria, *Streptococcus* spp., *Eikenella corrodens*, and *Staphylococcus aureus*, among others, have also been isolated [55,56,57,58,59,60]. In addition, circulating proteins with prothrombotic potential and specific virulence patterns in fusobacteria have been identified in Lemierre syndrome and may worsen prognosis [55,56].

Most published studies initiate broad-spectrum antibiotics, typically continued for 4–6 weeks, although there is no consensus on either duration or the optimal regimen. The most commonly used regimens include beta-lactams alone or combined with anaerobe-active antibiotics such as metronidazole or clindamycin [54,59]. In certain circumstances, coverage for resistant organisms such as methicillin-resistant *Staphylococcus aureus* (MRSA) may be required with vancomycin, linezolid, or other preferred agents [61,62,63].

The role of anticoagulation in preventing thrombus progression or septic embolization remains uncertain, as available evidence is limited to retrospective case series and no randomized controlled trials are currently available. In practice, most patients do not receive anticoagulation; however, some authors recommend it in the presence of thrombus progression, persistent fever, or persistent bacteremia after 5–7 days of appropriate antimicrobial therapy due to the risk of retrograde extension into the venous sinuses [64]. Optimal anticoagulation duration is not defined. In general, it is discontinued after clinical improvement and radiologic stabilization, although regimens exceeding 6 weeks have been described in the presence of septic emboli or associated prothrombotic factors [64]. A systematic review identified 1 case treated with rivaroxaban and 1 with edoxaban, both with favorable outcomes [64]. In exceptional cases of uncontrolled sepsis or persistent septic emboli despite appropriate antibiotic and anticoagulant therapy, ligation or excision of the vein has been performed [65].

### 5.5. Dural Venous Sinuses

**Figure 2:**
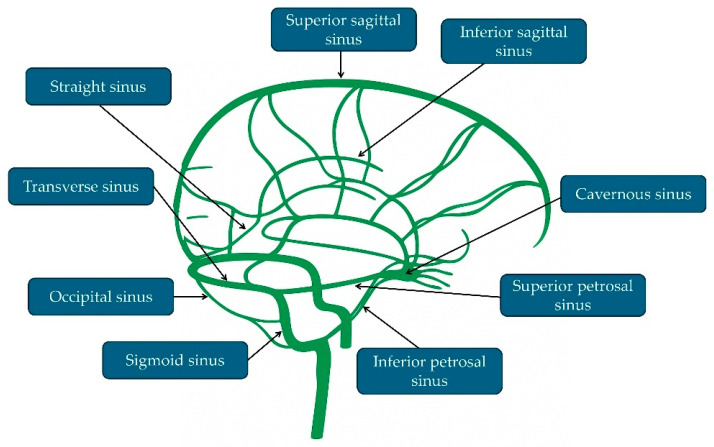
Anatomy of the dural venous sinuses.

The dural venous sinuses are channels located between the layers of the dura mater that collect cerebral venous blood and drain it into the internal jugular veins. Major sinuses include the superior sagittal, straight, transverse, sigmoid, and cavernous sinuses, the latter closely related to the skull base and adjacent neurovascular structures (Figure 2).

Septic dural venous sinus thrombosis is an uncommon but severe complication of otorhinolaryngologic and craniofacial infections. The cavernous sinus is most frequently involved. Management combines antimicrobial therapy to control the infectious source and, in some cases, anticoagulation. Infectious etiologies include bacterial and fungal pathogens, although cultures are negative in most cases. The most frequently implicated microorganisms are *Staphylococcus aureus* (particularly methicillin-resistant strains), other staphylococci, *Streptococcus* spp., anaerobes, and Gram-negative bacilli. In immunocompromised patients, fungal infections caused by *Rhizopus* spp., other mucormycosis agents, and *Aspergillus* spp. have also been reported [3,66].

Empiric antibiotic therapy should cover Gram-positive, Gram-negative, and anaerobic organisms, especially in odontogenic or sinus-origin cases. Proposed regimens include vancomycin plus cefepime (particularly when *Pseudomonas aeruginosa* is suspected) and metronidazole for anaerobic coverage or vancomycin plus meropenem [66,67]. There is no consensus on the optimal duration; however, most authors recommend at least 3 weeks, with discontinuation individualized based on the clinical course [67].

The role of anticoagulation in septic cavernous sinus thrombosis is controversial. No randomized controlled trials support its use; evidence derives from case series and retrospective studies. Southwick et al. reviewed 86 cases reported between 1940 and 1984 and observed lower mortality among patients treated with UFH compared with those not anticoagulated (14% vs. 36%) [68]. Subsequently, a retrospective study analyzed 88 published cases from 1941 to 1988. Mortality was 13% in patients treated with UFH versus 24% in those treated with antibiotics alone. Although this difference did not reach statistical significance, early anticoagulation was associated with better neurologic outcomes, including lower rates of blindness, stroke, ophthalmoplegia, and epilepsy [69]. Weerasinghe and Lueck analyzed 88 patients with septic cavernous sinus thrombosis reported between 1980 and 2015 [66]. In that series, 47% received anticoagulation with UFH, LMWH, or dalteparin for periods ranging from 2 weeks to more than 3 months. Anticoagulated patients had a higher likelihood of complete recovery (53.6% vs. 32%) and lower mortality (12% vs. 28%). Across studies, anticoagulation was not associated with an increased intracranial hemorrhage risk, even in patients with hemorrhagic infarction. Optimal duration remains uncertain. Most authors recommend anticoagulation for at least 4–6 weeks and up to 3 months.

Anticoagulation is not routinely part of the management of septic lateral sinus thrombosis (formed by the transverse and sigmoid sinuses). It is generally reserved for cases with thrombus progression despite antibiotics and surgery, or for those with documented thrombophilia. This approach is supported by the observation that infectious lateral sinus thrombosis often resolves with antibiotics and may recanalize spontaneously [70].

**Figure 3:**
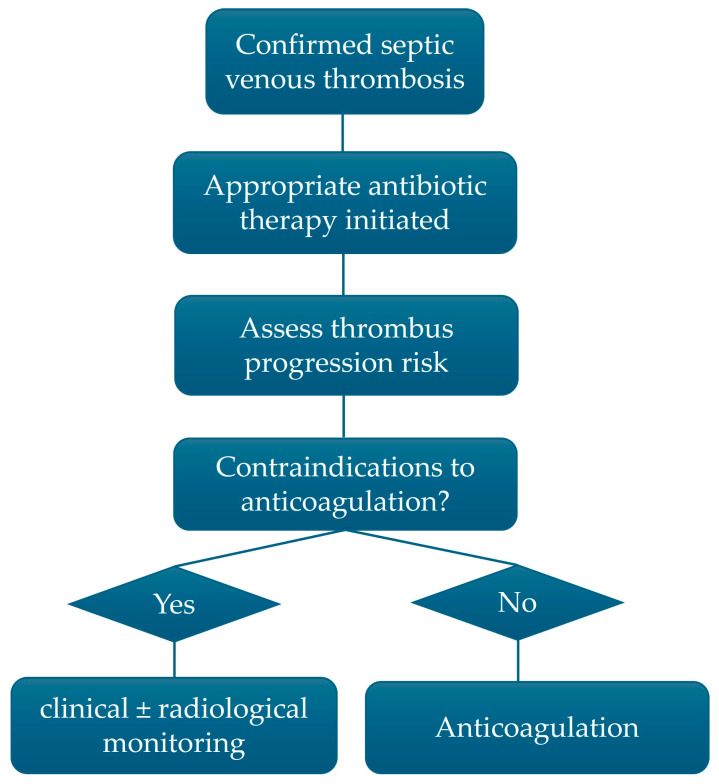
Proposed clinical algorithm for the management of septic venous thrombosis.

Antimicrobial treatment recommendations for SVT by location are summarized in Table 1, and anticoagulation recommendations are presented in Table 2. Figure 3 presents a pragmatic clinical algorithm for the management of DVT, based on infection control, thrombotic risk stratification, and hemorrhage risk assessment.

## 6. Conclusions

Septic venous thrombosis is an uncommon but serious complication of multiple infectious processes and carries substantial morbidity and mortality. Clinical presentation varies by location and primary source, and early diagnosis with prompt initiation of antimicrobial therapy is essential to improve outcomes. However, current evidence regarding optimal antibiotic duration, the most appropriate regimen, and the role of anticoagulation remains limited, relying primarily on case series, retrospective studies, and expert recommendations. Therefore, clinical management should be individualized within a multidisciplinary framework. Future priorities include randomized clinical trials and well-designed prospective studies to more precisely define unresolved areas of uncertainty. In addition, it is critical to evaluate the efficacy of newer antimicrobial and antifungal agents in multidrug-resistant settings.

## Figures and Tables

**Table 1:** Antimicrobial treatment recommendations for septic venous thrombosis by location.

Location	Most Frequent Microorganisms	Recommended Antimicrobial Treatment	Suggested Duration
Portal vein and its branches	*Bacteroides* spp., *Escherichia coli*, *Streptococcus* spp. Polymicrobial in ~30%.	Third-generation cephalosporin or fluoroquinolone plus metronidazole; monotherapy with a beta-lactamase inhibitor or a carbapenem if there is a risk of multidrug resistance.	4–6 weeks
Pelvic veins	*Streptococcus* spp., enteric Gram-negative bacteria, and anaerobes. Cultures frequently negative.	Gentamicin plus clindamycin (traditional use); currently, monotherapy with a beta-lactam/beta-lactamase inhibitor or a cephalosporin/fluoroquinolone plus an anaerobe-active agent (clindamycin preferred during breastfeeding).	48–72 h after fever resolution and clinical stabilization
Catheter-associated venous thrombosis	*Staphylococcus aureus*, coagulase-negative *Staphylococcus*, *Streptococcus* spp., enteric Gram-negative bacteria, *Pseudomonas aeruginosa*, and *Candida* spp.	Vancomycin or daptomycin for Gram-positive coverage; add piperacillin–tazobactam, carbapenem, fourth-generation cephalosporin, fluoroquinolone, or aminoglycoside if Gram-negative infection is suspected; empiric antifungal therapy if *Candida* risk factors are present.	Traditionally 3–6 weeks; recent studies suggest <21 days in selected cases
Internal jugular vein	*Fusobacterium necrophorum* (most frequent), other *Fusobacterium* spp., enteric Gram-negative bacteria, *Streptococcus* spp., *Eikenella corrodens*, and *Staphylococcus aureus*.	Beta-lactam monotherapy or combined with metronidazole or clindamycin; add anti-MRSA coverage (vancomycin, linezolid) in selected cases.	4–6 weeks
Dural venous sinuses	*Staphylococcus aureus* (including MRSA), *Streptococcus* spp., other staphylococci, anaerobes, Gram-negative bacilli; in immunocompromised patients, *Rhizopus* spp., *Aspergillus* spp.	Vancomycin plus cefepime (if *Pseudomonas aeruginosa* is suspected) plus metronidazole; alternative: vancomycin plus meropenem. Add empiric antifungal therapy if invasive fungal infection is suspected.	

Abbreviations: MRSA, methicillin-resistant *Staphylococcus aureus*.

**Table 2:** Anticoagulation recommendations for septic venous thrombosis by location.

Location	Available Evidence	Anticoagulation Recommendations	Suggested Duration
Portal vein and its branches	Case series and retrospective studies. Higher recanalization rates and lower risk of portal hypertension.	May be considered in thrombus progression, persistent fever/bacteremia, mesenteric extension, or underlying prothrombotic conditions. DOACs use in non-septic portal vein thrombosis shows favorable results.	Individualized based on clinical and radiologic course. From 2–4 weeks up to 3 months
Pelvic veins	Case series. High rates of clinical resolution with heparin.	Recommended, particularly in ovarian vein involvement, iliac or caval extension, or septic emboli.	2–6 weeks; extend if major vessel involvement or embolization
Catheter-associated venous thrombosis	Heterogeneous evidence; observational studies.	Consider in combination with catheter removal and antibiotics; some studies suggest improved outcomes.	Not defined; individualized based on extent and risk factors
Internal jugular vein	Retrospective series and isolated reports.	Not routine. May be considered in progression, persistent fever/bacteremia, or extension toward venous sinuses. Isolated DOACs use with favorable outcomes reported.	Not defined; generally until clinical/radiologic stabilization; >6 weeks in selected cases
Dural venous sinuses	Retrospective series and case reports.	May be considered in most cases. Evidence suggests clinical benefit without increased hemorrhagic risk. Not routinely recommended for lateral sinus thrombosis.	Not defined; individualized. Similar to non-septic thrombosis: 4–12 weeks

Abbreviations: DOACs, direct oral anticoagulants.

## Data Availability

The data supporting this study’s findings are available from the corresponding author.

## Abbreviations

The following abbreviations are used in this manuscript:

CT	Computed tomography
DIC	Disseminated intravascular coagulation
DOACs	Direct oral anticoagulants
ICU	Intensive care unit
LMWH	Low-molecular-weight heparin
MRI	Magnetic resonance imaging
PVT	Portal vein thrombosis
SSC	Surviving Sepsis Campaign
SVT	Splanchnic vein thrombosis
UFH	Unfractionated heparin
VTE	Venous thromboembolism
